# Preoperative Predictors for 90-Day Mortality after Pancreaticoduodenectomy in Patients with Adenocarcinoma of the Ampulla of Vater: A Single-Centre Retrospective Cohort Study

**DOI:** 10.1155/2021/6682935

**Published:** 2021-02-27

**Authors:** Ramiro Fernandez-Placencia, Francisco Berrospi-Espinoza, Karla Uribe-Rivera, Jose Medina-Cana, Ivan Chavez-Passiuri, Nestor Sanchez-Bartra, Kori Paredes-Galvez, Carlos Luque-Vasquez Vasquez, Juan Celis-Zapata, Eloy Ruiz-Figueroa

**Affiliations:** ^1^Hepato-Pancreato-Biliary Section, Department of Abdominal Surgery, Instituto Nacional de Enfermedades Neoplasicas (INEN), Surquillo, Peru; ^2^Department of Abdominal Surgery, Instituto Nacional de Enfermedades Neoplasicas (INEN), Surquillo, Peru; ^3^Department of Anesthesiology, Instituto Nacional de Enfermedades Neoplasicas (INEN), Surquillo, Peru

## Abstract

**Background:**

The standard treatment for ampullary adenocarcinoma is pancreaticoduodenectomy. Identification of preoperative risk factors might help the clinician to select patients fit for resection and potentially decrease morbidity and mortality after PD. We conducted a cohort study to determine the preoperative factors related to 90-day severe morbidity and mortality after PD.

**Methods:**

We conducted a retrospective cohort study in patients with a diagnosis of ampullary adenocarcinoma who underwent an open PD between January 2010 and December 2019 at our tertiary centre.

**Results:**

Independent preoperative predictors of mortality were the albumin-bilirubin (ALBI) grade 3 (OR: 21.7; CI 95: 2.1–226.9; *p*=0.01) and the estimated glomerular filtration rate (eGFR) <90 mL/min/1.73 m^2^ (OR: 17.7; CI 95: 1.8–172.6; *p*=0.013). The eGFR <90 mL/min/1.73 m^2^ (OR = 6.6; CI 95: 1.9–23.4; *p*=0.003) and prothrombin time (OR = 1.5; CI 95; 1.1–2.1; *p*=0.005) were independent predictors for severe morbidity.

**Conclusion:**

These findings suggest that baseline renal function measured by the eGFR and liver function categorized with the ALBI grading are predictors of severe morbidity and mortality. Thus, they should be considered when selecting patients for PD or the use of neoadjuvant treatments. Further research is warranted.

## 1. Introduction

The adenocarcinoma of the ampulla of Vater is less frequent than pancreatic ductal adenocarcinoma (PDAC) [1, 2] with an incidence of 0.59 cases per 100,000 individuals [3]. A pancreaticoduodenectomy (PD) is the procedure of choice with curative intent [4] but the risk of pancreatic fistula is different from that of PDAC, mainly due to the characteristics of the pancreas remnant [5, 6]. Different pancreatic anastomoses have been described but none have shown superiority in terms of mortality [7, 8], which partially explains the wide variability in reconstruction techniques and practices around the world [9]. Despite mortality rates after PD having decreased to less than 5% in many centres [10, 11], others experience higher mortality rates after resection of the ampullary adenocarcinoma [12, 13].

Persistent jaundice has been implicated as an important risk factor for dismal results after PD [14, 15]. Accordingly, the selective use of preoperative biliary stenting is recommended [16, 17] after assessment of the clinical status [18] and serum bilirubin levels. For the latter, different thresholds have been established: 150 *µ*mol/L [17], 250 *µ*mol/L [19], and 300 *µ*mol/L [14]. Not limited to the aforementioned, other perioperative factors such as age, cardiac surgery, hypertension, use of steroids [20], sex, operative time, and 24-hour urine output [21] are part of scoring systems that help the prediction of outcomes after PD is performed for different neoplasms. In patients with ampullary adenocarcinoma, one study reported that age (>75 years), positive blood culture, and serum albumin levels (<3.0 g/dL) were predictors of mortality [11].

Recently, the albumin-bilirubin (ALBI) grade has been used as an objective parameter to estimate liver function and predict morbidity and mortality after hepatectomy [22–25]. A previous study of patients with miscellaneous aetiologies who underwent PD could not demonstrate a predictive role [26]. Preoperative renal function, measured by the estimated glomerular filtration rate (eGFR), has been also proposed as a predictor of complications after pancreatic resection [27].

Timely identification of risk factors might help the clinician in patient selection and potentially decrease morbidity and mortality after PD in patients with ampullary adenocarcinoma. We conducted a cohort study to specifically assess the preoperative factors related to 90-day mortality and severe morbidity after PD in patients with adenocarcinoma of the ampulla of Vater at our centre.

## 2. Methods

### 2.1. Study Design and Patient Selection

We conducted a retrospective cohort study in patients with a diagnosis of ampullary adenocarcinoma who underwent an open PD between January 2010 and December 2019 at our tertiary centre. Demographic, clinical, and surgical variables were prospectively collected in our clinical database. Patients with other ampullary neoplasms were not included in the analysis. We specifically analysed the ALBI grade and the eGFR. Blood samples were obtained during the week before the index procedure as part of the routine testing. The ALBI grade and the eGFR were obtained retrospectively by one investigator (RF). Postoperative data were collected until postoperative day (POD) 90, and the last follow-up was recorded in May 2020. The Institutional Review Board approved the study in accordance with the Declaration of Helsinki [28].

All procedures were performed by an expert group of hepatopancreatobiliary surgeons from our centre. Determined on a case-by-case decision during multidisciplinary team (MDT) meetings, patients were considered generally unsuitable for surgery when any of the following items were found: poor performance status (ECOG ≥2), M1 disease (according to TNM staging), and bilirubin levels ≥300 mmol/L. In rare and selected cases, the latter threshold could have been crossed after MDT consensus. When bilirubin levels were above this upper limit or the patient had cholangitis, preoperative biliary stenting was indicated and PD was carried out after 4–6 weeks based on previous reassessment.

### 2.2. Surgical Technique

In brief, the operative technique entailed putting the patient in a supine position and beginning with a midline incision and inspection for metastases to other organs. Then a Kocher manoeuvre was performed to expose the posterior aspect of the duodenum and pancreatic head. Thereafter, the procedure was continued by mobilization of the proximal jejunum and the angle of Treitz, followed by the ligation and division of corresponding jejunal vessels. For ampullary adenocarcinomas, we performed a level 2 mesopancreatic resection [29]. The type of pancreaticojejunostomy (PJ) included a modified dunking [30], Blumgart et al. [31], and the classic duct-to-mucosa PJ [32]. The use of an external stent in the pancreatic duct corresponded to the surgeon's preference. Two Blake drains were placed around the PJ in all cases. Prophylactic octreotide was not part of our practice.

### 2.3. Endpoints and Definitions

The primary endpoint was to determine predictors of 90-day mortality after PD for ampullary adenocarcinoma. The secondary endpoint was to identify predictors of severe morbidity (>IIIb) according to the Dindo-Clavien classification [33].

Clinically relevant postoperative pancreatic fistula (CR-POPF), postpancreatectomy haemorrhage (PPH), biliary fistula, and delayed gastric emptying (DGE) were defined according to the International Study Group in Pancreatic Surgery (ISGPS) [34, 35].

#### 2.3.1. ALBI Grading

The ALBI grading is an ordinal measurement derived from a regression model that utilises the serum values of albumin and bilirubin and transforms them into a score, in order to assess liver function and estimate the survival of patients with hepatocellular carcinoma [24, 36]. Also, other aetiologies (i.e., Primary Biliary Cirrhosis) [37] and clinical outcomes (i.e., after liver resection) have been reported [22, 38]. The ALBI grading was determined in a retrospective fashion using the preoperative albumin and bilirubin values (obtained during the week before the index procedure) and calculated online (https://www.mdcalc.com/albi-albumin-bilirubin-grade-hepatocellular-carcinoma-hcc).

#### 2.3.2. Glomerular Filtration Rate

To further assess the renal function, we used the eGFR [39, 40]. In the present study, it was determined using the Modification of Diet in Renal Disease (MDRD) formula as performed by Squires et al. [27]; the cut-off used to consider normal function was 90 mL/min/1.73 m^2^. The eGFR grading was determined in a retrospective fashion using the preoperative serum creatinine levels (obtained during the week before the index procedure) and calculated online (https://www.mdcalc.com/mdrd-gfr-equation).

### 2.4. Follow-Up

Postoperative follow-up was recorded until POD90 as this is a more consistent measure in hepatopancreatobiliary surgeries [41, 42]. After discharge, patients were scheduled to have an outpatient visit on POD15, POD30, and POD90. Any unfavourable event was recorded during this period.

### 2.5. Statistical Analysis

The continuous variables were reported as median (IQR) and categorical variables as counts (percentages). For the univariate analysis, the Mann–Whitney *U*-test was used for continuous variables; categorical variables were compared using the chi-squared test, or Fisher's exact test when appropriate. Clinically relevant variables with a *p* < 0.1 in univariate analysis were introduced into a binary logistic regression model to identify predictors of severe morbidity and mortality. Significance was set at *p* < 0.05 in multivariable analysis. Statistical analysis was performed using IBM SPSS v.25 (IBM Corp., Armonk, NY).

## 3. Results

### 3.1. Preoperative Characteristics

**Figure 1:**
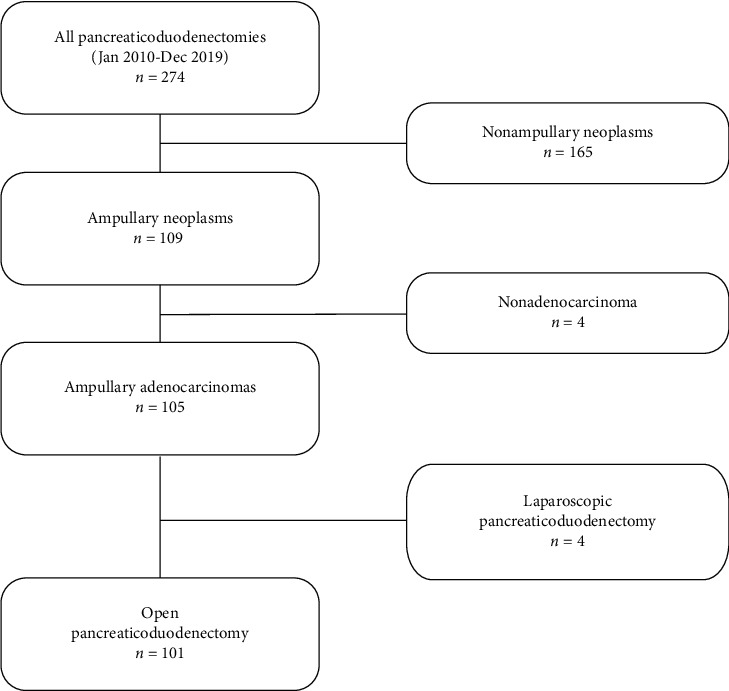
Patient selection flow chart.

101 patients were eligible for analysis (Figure 1). In three patients, the ALBI grade could not be assessed due to missing data. Preoperative patient characteristics are shown in Table 1.

### 3.2. Operative Findings and Pathologic and Clinical Outcomes

None of the patients were suspected for cirrhosis and neither had abnormalities reported on the liver surface. The median diameter of the pancreatic duct was 5 mm (3–7). In 27 cases, the pancreatic characteristics could not be retrieved. The median (IQR) fistula risk score (5) was 3 (2–5) (Table 2).

The median tumour size was 27.5 mm and the majority of patients (79%) presented the intestinal type. Perineural invasion was 33% and microscopic vascular invasion was 38%.

After surgical resection, 40 patients had a clinically relevant POPF and 28 patients had a PPH [A: (*n* = 5), B (*n* = 18), and C (*n* = 10)]. Overall morbidity was 83% and severe morbidity was observed in 20 patients. PPH (*n* = 15), CR-POPF (*n* = 15), respiratory failure (*n* = 3), and acute wound dehiscence (*n* = 1) accounted for most common severe complications.

Postoperative mortality occurred in 11 patients. Causes of death were related to CR-POPF (type C: 5 patients) and PPH (type C: 6 patients).

### 3.3. Predictors of Mortality

**Figure 2:**
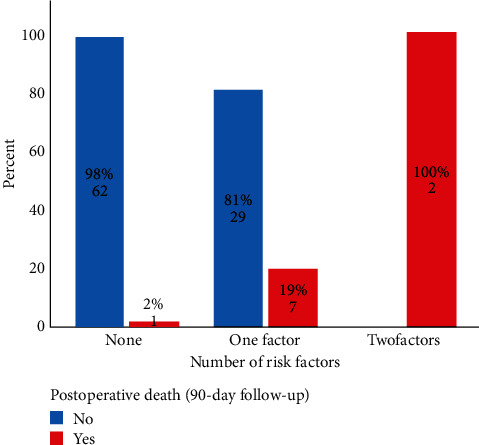
Postoperative death rate according to the number of risk factors.

After univariate analysis, age (*p*=0.005), ALBI grade (*p*=0.01), ASA score (*p*=0.024), eGFR <90 mL/min/1.73 m^2^(*p*=0.015), preoperative RBC packs transfusion (*p*=0.031), serum creatinine (*p*=0.028), and serum albumin levels (*p*=0.038) were selected for multivariable analysis. Independent preoperative predictors of mortality were the ALBI grade 3 (OR: 21.7; CI 95: 2.1–226.9 (*p*=0.01)) and the eGFR <90 mL/min/1.73 m^2^ (OR: 17.7; CI 95: 1.8–172.6 (*p*=0.013)) (Table 3). Subgroups were organized according to the number of predictors (Figure 2).

### 3.4. Predictors of Severe Morbidity

**Figure 3:**
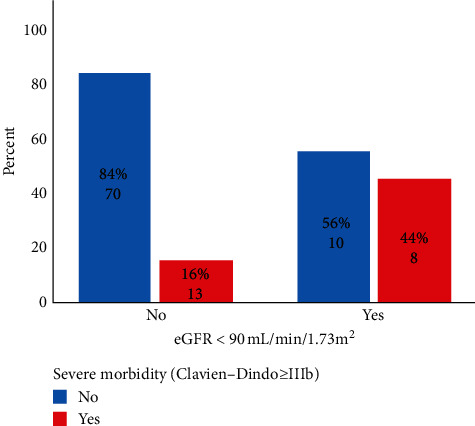
Severe morbidity (Clavien–Dindo ≥IIIb) rate after PD grouped by having an eGFR<90 mL/min/1.73 m^2^.

In the univariate analysis, eGFR <90 mL/min/1.73 m^2^(*p*=0.011), prothrombin time (*p*=0.012), INR (*p*=0.072), serum glucose (*p*=0.074), and serum creatinine (*p*=0.008) were fit for multivariable analysis. Then eGFR <90 mL/min/1.73 m^2^ (OR = 6.6; CI 95: 1.9–23.4 (*p*=0.003)) and prothrombin time (OR = 1.5; CI 95: 1.1–2.1 (*p*=0.005)) were independent predictors for this outcome. When grouped according to the eGFR (Figure 3), the rate of severe morbidity increased from 16 to 44% (Table 4).

## 4. Discussion

According to the present results, an ALBI 3 grade and an eGFR <90 mL/min/1.73 m^2^ are important preoperative predictors of mortality and morbidity after PD in ampullary adenocarcinoma. It is noteworthy that patients who had neither of these risk factors had a 2% chance of postoperative mortality.

This study shows data of a selected and prospectively followed up group of patients with adenocarcinoma of the ampulla of Vater who were treated in a comprehensive tertiary centre with a high volume of PD.

Despite a careful patient selection and the use of preoperative biliary stenting in selected cases with serum bilirubin levels ≥300 mmol/L as a cut-off, we had a higher mortality rate compared to PDAC (4.8%, unpublished data). This justified the search for other preoperative predictors of mortality.

Inherent limitations from a retrospective single-centre study were noticed, the first being related to the small sample and the number of events of interest from this population. Another drawback is that the ALBI grades could not be retrieved in three patients.

Several high-quality studies demonstrate no differences in morbidity and mortality for PJ reconstruction types [43, 44]; this also was reflected in our population (supplementary table) despite the fact that ampullary adenocarcinomas have an increased rate of high-risk features for CR-POPF (soft pancreas, pancreatic duct ≤5 mm) [45].

The ALBI grade has been widely used in hepatocellular carcinoma and liver surgery. In case of patients who underwent PD, Sandini et al. [26] could not identify the ALBI grade of prognostic relevance, probably because all the patients included in the study were part of a selected cohort in which all patients had preoperative biliary stenting. As previously reported by Lai et al., patients with hypoalbuminemia, undernourishment, cholangitis, jaundice-induced liver, or renal failure are ideal candidates for preoperative biliary stenting [18].

Renal function impairment has been associated with complications after pancreatic surgery [27]. In the present study, the eGFR <90 mL/min/1.73 m^2^ showed a 2.75-fold increase in the risk of severe morbidity and was also a predictor of mortality. Although infrequent in our population, both risk factors conferred a 100% mortality in our cohort; this depicts the organ dysfunction not identified by standard methods (i.e., serum creatinine, albumin, and bilirubin).

Perioperative red blood cell (RBC) transfusions have been identified as prognostic factors for overall survival [46, 47], postoperative morbidity [48], and mortality [49] after PD, probably due to immune function impairment. However, in our study, the use of preoperative RBC transfusions did not reach statistical significance for mortality or severe morbidity. Therefore, it could be hypothesized that intraoperative or postoperative RBC transfusions have a more deleterious effect. We did not evaluate the perioperative transfusion as our model tried to identify factors before the decision to perform PD was made (patient selection).

These findings suggest that baseline renal function measured by the eGFR and liver function categorized with the ALBI grading are predictors of severe morbidity and mortality. We hypothesize that these conditions make the patient prone to severe morbidity or mortality if CR-POPF, PPH, or both occur. Standard bilirubin thresholds should be complemented with the ALBI grade in order to indicate preoperative biliary stenting and thus improve the clinical condition. The eGFR should be used as a screening method to assess the severity and chronicity of kidney disease and thus provide adequate perioperative care. If the clinical condition of these patients does not improve after preconditioning measures, PD should exhaust all the available strategies to mitigate the pancreatic fistula and minimize postpancreatectomy haemorrhage occurrence and severity provided the patient has been informed and accepted the higher risk associated. Otherwise, the use of less aggressive procedures (i.e., ampullectomy) could offer a probability of cure in this high-risk population with early ampullary adenocarcinomas.

The use of neoadjuvant treatments (i.e., chemoradiotherapy or chemotherapy) could mean a bridge to surgery while improving the clinical condition. Cloyd et al. [50] demonstrated that, with this approach, the overall survival after PD in this population did not seem to worsen.

To the best of our knowledge, this is the first study to have presented the ALBI grade as a predictor of mortality after PD. Further studies are warranted, especially in centres where ampullary adenocarcinomas are more frequent than pancreatic adenocarcinoma, as in the case of our institution.

## Figures and Tables

**Table 1:** Clinical, laboratory, and operative patient characteristics.

	*n* = 101 (100%)
*Patient demographics*	
Age (y), median (IQR)	60 (52–67)
Sex, male : female (count, %)	47 (46.5):54 (53.5)
Body mass index (kg/m²), median (IQR)	24.6 (21.7–27.5)
MELD score, median (IQR)	9 (7–14)
eGFR <90 mL/min/1.73 m², count (%)	18 (17.8)

*ALBI score (count, %)*	
ALBI 1 score	30 (29.7)
ALBI 2 score	46 (45.5)
ALBI 3 score	22 (21.8)
Unknown	3 (3)

*ASA score (count, %)*	
ASA I	2 (2)
ASA II	74 (73.3)
ASA III	25 (24.8)

*Past medical history*	
Preoperative biliary drainage, count (%)	29 (28.7)
Endoscopic	18
Percutaneous	6
Surgical	5
Previous history of cholangitis, count (%)	8 (7.9)
Number of RBC packs transfused^†^, median (IQR)	0 (0-1)

*Preoperative laboratory tests*	
Haemoglobin (g/L), median (IQR)	115 (107–126)
Platelet count (10^9^/L), median (IQR)	285 (242–381)
International Normalized Ratio, median (IQR)	1.07 (1.02–1.16)
Prothrombin time (sec), median (IQR)	12.9 (11.8–14.5)
Serum total bilirubin (*µ*mol/L), median (IQR)	22.1 (12–101.4)
Serum glucose (*µ*mol/L), median (IQR)	5.1 (4.7–5.6)
Serum creatinine (*µ*mol/L), median (IQR)	55 (47–66)
Serum albumin (g/L), median (IQR)	37 (32–41)
Serum CA 19-9 (IU/mL), median (IQR)	22.2 (9.7–91.4)

Continuous data are expressed as median (IQR). Categorical data are expressed as count (%). ^†^Preoperative transfusion.

**Table 2:** Operative and pathologic patient characteristics.

	*n* = 101 (100%)
*Intraoperative variables*	
*Pancreas texture, count (%)*	
Soft pancreas	48 (47.5)
Hard pancreas	26 (25.7)
*PJ type*	
Modified dunking	50 (49.5)
Blumgart	37 (36.6)
Classic duct-to-mucosa	14 (13.9)
Operative time in min, median (IQR)	370 (305–435)
Estimated blood loss in cc, median (IQR)	300 (200–450)

*Pathology findings*	
*Tumour subtype*	
Intestinal	79 (78.2)
Pancreatobiliary	14 (13.9)
Others	8 (7.9)
Maximum diameter in cm, median (IQR)	27.5 (18–40)
Lymph node invasion, count (%)	41 (40.6)
Number of lymph nodes retrieved, median (IQR)	17 (12–23)
*Resection margin status*	
R0	100 (99)
R1	1 (1)

*Postoperative course*	
Hospitalization length of stay (days), median (IQR)	12 (9–16)
Severe morbidity (≥IIIb), count (%)	21 (20.8)
*CR-POPF (ISGPS), count (%)*	
Type B	33 (33)
Type C	7 (7)
*PPH (ISGPS A, B, and C), count (%)*	
Type A	5 (5)
Type B	18 (17.8)
Type C	10 (9.9)
Delayed gastric emptying (ISGPS A, B, and C), count (%)	2 (2)
Postoperative death (90 days), count (%)	11 (10.5)

Continuous data are expressed as median (range). Categorical data are expressed as count (%).

**Table 3:** Univariate and multivariable analysis for prediction of 90-day mortality after pancreaticoduodenectomy for ampullary adenocarcinoma.

	Mortality		Univariate		Multivariable		
	No (*n* = 91)	Yes (*n* = 10)	*p* value	*p* value	OR	CI 95	
Age (years), mean (SD)	58.7 (11.3)	68.8 (6)	*0.005* ^*φ*^	0.077	1.1	0.99	1.2
Male sex, count (%)	41 (45.1)	6 (6)	0.508^*χ*^				
Body mass index (kg/m^2^), mean (SD)	24.9 (4.4)	25.3 (3.1)	0.737^*φ*^				
MELD score, mean (SD)	10.5 (3.9)	12.7 (5.9)	0.225^*φ*^				
ALBI grade, count (%)			*0.010* ^*χ*^				
ALBI 1	29 (31.9)	1 (10)		0.037			
ALBI 2	43 (47.3)	3 (30)		0.383	3.05	0.25	37.5
ALBI 3	16 (17.6)	6 (60)		**0.01**	**21.7**	**2.1**	**226.9**
Undetermined	3 (3.2)	0					
ASA score, count (%)			*0.024* ^*χ*^	0.385			
ASA I	2 (2.2)	0					
ASA II	70 (76.9)	4 (40)					
ASA III	19 (20.9)	6 (60)					
eGFR <90 mL/min/1.73 m^2^, count (%)	13 (14.3)	5 (50)	*0.015* ^*χ*^	**0.013**	**17.7**	**1.8**	**172.6**
Preoperative RBC transfusion, count (%)	23 (25.3)	6 (60)	*0.031* ^*χ*^	0.322			
Preoperative biliary drainage, count (%)	27 (29.7)	0	0.720^*χ*^				
Haemoglobin in g/L, mean (SD)^†^	116.2 (14.2)	119.6 (19.1)	0.878^*φ*^				
Platelet count in 10^9^/L, mean (SD)^†^	336.4 (156)	282 (89.3)	0.403^*φ*^				
International Normalized Ratio, mean (SD)^†^	1.09 (0.1)	1.21 (0.3)	0.202^*φ*^				
Prothrombin time, mean (SD)^†^	13 (1.7)	14.4 (2.8)	0.114^*φ*^				
Serum total bilirubin in *µ*mol/L, mean (SD)^†^	61.8 (79.7)	98 (107.7)	0.437^*φ*^				
Serum creatinine in *µ*mol/L, mean (SD)^†^	57 (14.7)	70.4 (20.9)	0.028^*χ*^	0.223			
Serum glucose in *µ*mol/L, mean (SD)^†^	5.5 (1.8)	4.97 (0.4)	0.322^*φ*^				
Serum albumin in g/L, mean (SD)^†^	36.6 (5.8)	30.6 (10.4)	*0.038* ^*φ*^	0.291			

^*φ*^ *χ*Mann–Whitney *U*-test; ^*χ*^chi-square or Fisher's exact test; ^†^preoperative laboratory values. Variables with a *p* < 0.1 (italics) in the univariate analysis were used for multivariable analysis. Significance was set at *p* < 0.05 (marked in bold).

**Table 4:** Univariate and multivariable analysis for prediction of 90-day severe morbidity (Clavien–Dindo ≥IIIb) after pancreaticoduodenectomy for ampullary adenocarcinoma.

	Severe morbidity		Univariate		Multivariable		
	No (*n* = 80)	Yes (*n* = 21)	*p* value	*p* value	OR	CI 95	
Age, mean ± SD	59 (10.8)	62.5 (12.9)	*0.141* ^*φ*^				
Male sex, count (%)	35 (43.8)	12 (57.1)	0.273^*χ*^				
Body mass index (kg/m^2^), mean ± SD	24.6 (4.2)	26.1 (4.7)	0.322^*φ*^				
MELD score, mean ± SD	10.4 (3.9)	11.8 (4.8)	0.584^*φ*^				
ALBI grade, count (%)			0.653^*χ*^				
ALBI 1	25 (31.3)	5 (23.8)					
ALBI 2	36 (45)	10 (47.6)					
ALBI 3	16 (20)	6 (28.6)					
Undetermined	3 (3.7)	0					
ASA score, count (%)			0.479^*χ*^				
ASA I	2 (2.5)	0					
ASA II	60 (75)	14 (66.7)					
ASA III	18 (22.5)	7 (33.3)					
eGFR <90 mL/min/1.73 m^2^, count (%)	10 (12.5)	8 (38.1)	*0.011* ^*χ*^	**0.003**	**6.6**	**1.9**	**23.4**
Preoperative RBC transfusion, count (%)	22 (27.5)	7 (33.3)	0.599^*χ*^				
Preoperative biliary drainage, count (%)	23 (28.7)	6 (28.6)	0.987^*χ*^				
Haemoglobin in g/L, mean ± SD^†^	116.6 (14)	116.5 (17.5)	0.487^*φ*^				
Platelet count in 10^9^/L, mean ± SD^†^	339.1 (162)	300 (90)	0.682^*φ*^				
International Normalized Ratio, mean ± SD^†^	1.08 (0.1)	1.17 (0.2)	*0.072* ^*φ*^	0.800			
Prothrombin time, mean ± SD^†^	12.9 (1.7)	14.2 (2.2)	*0.012* ^*φ*^	**0.005**	**1.5**	**1.1**	**2.1**
Serum total bilirubin in *µ*mol/L, mean ± SD^†^	61.7 (78.2)	80.1 (100.7)	0.741^*φ*^				
Serum creatinine in *µ*mol/L, mean ± SD^†^	56.3 (14.6)	66.6 (17.9)	*0.008* ^*χ*^	0.417			
Serum glucose in *µ*mol/L, mean ± SD^†^	5.5 (1.9)	4.9 (0.5)	*0.074* ^*φ*^	0.245	0.6	0.3	1.4
Serum albumin in g/L, mean ± SD^†^	36.3 (6.2)	34.7 (7.6)	0.361^*φ*^				

^*φ*^Mann–Whitney *U*-test; ^*χ*^chi-square or Fisher's exact test; ^†^preoperative laboratory values. Variables with a *p* < 0.1 (italics) in the univariate analysis were used for multivariable analysis. Significance was set at *p* < 0.05 (marked in bold).

## Data Availability

The data used to support the findings of this study have not been made available because of institutional policies.

## Supplementary Materials

Supplementary Materials Table: comparison of intraoperative factors associated with severe morbidity and mortality.
