# Supplementary material for: Preoperative Predictors for 90-Day Mortality after Pancreaticoduodenectomy in Patients with Adenocarcinoma of the Ampulla of Vater: A Single-Centre Retrospective Cohort Study
Source: Surg Res Pract. 2021 Feb 27;2021:6682935. doi: 10.1155/2021/6682935 (PMC7937469; doi:10.1155/2021/6682935)
Supplement: Supplementary Materials — Table: comparison of intraoperative factors associated with severe morbidity and mortality. [file 6682935.f1.docx]

| **Supplementary Table : Comparison of severe morbidity and mortality according intraoperative factors** | | | | | | | | | |
| --- | --- | --- | --- | --- | --- | --- | --- | --- | --- |
|  |  |  |  |  | Severe Morbidity |  |  | Postoperative mortality | |
|  |  |  |  |  |  |  |  |  |  |
|  |  |  |  |  | p-value |  |  | p-value | |
|  |  |  |  |  |  |  |  |  |  |
| Operative time in min. | | | |  | **0.003ᵠ** |  |  | 0.309ᵠ | |
| Pancreaticojejunostomy type | | |  |  | 0.939ᵡ |  |  | 0.804ᵡ | |
| Estimated blood loss in cc. | | | |  | 0.446ᵠ |  |  | 0.558ᵠ | |
| Gland texture (soft) | | |  |  | 0.442ᵡ |  |  | **0.047ᵡ** | |
| Main pancreatic duct, in mm. | | |  |  | 0.200ᵠ |  |  | 0.909ᵠ | |
|  | |  |  |  |  |  |  |  |  |
| ᵠ Mann-Whitney U test; ᵡ chi-square or Fisher's exact test | | | | | |  |  |  |  |
